# Cortisol levels in unmedicated patients with unipolar and bipolar major depression using hair and saliva specimens

**DOI:** 10.1186/s40345-020-0180-x

**Published:** 2020-03-05

**Authors:** Andrés Herane-Vives, Danilo Arnone, Valeria de Angel, Andrew Papadopoulos, Toby Wise, Luis Alameda, Kia-Chong Chua, Allan H. Young, Anthony J. Cleare

**Affiliations:** 1 Centre for Affective Disorders, Department of Psychological Medicine, Institute of Psychiatry, Psychology & Neuroscience, King’s College London, 103 Denmark Hill, London, SE5 8AF UK; 2 Neuroscience and Mental Health Group, Institute of Cognitive Neuroscience, University College London, London, UK; 3 Department of Psychiatry, College of Medicine and Health Sciences, United Arab Emirates University, Abu Dhabi, United Arab Emirates; 4 Department of Psychosis Studies, Institute of Psychiatry, Psychology & Neuroscience, King’s College London, London, UK; 5 Unit for Research in Schizophrenia, Center for Psychiatric Neuroscience, Department of Psychiatry, Lausanne University Hospital (CHUV), Lausanne, Switzerland

## Abstract

**Background:**

Differentiating between unipolar and bipolar depression can be clinically challenging, especially at first presentation. Patterns of cortisol secretion could aid diagnostic discrimination in affective disorders although there has been little comparative research to date. In this study, we investigated acute (saliva) and chronic (hair) cortisol levels concurrently in unmedicated unipolar and bipolar disorders by using conventional diagnostic criteria and self-report measures.

**Methods:**

Patients with unipolar and bipolar major depression and healthy controls were recruited and assessed. Cortisol levels were extracted from saliva and hair specimens. Depressive features were investigated according to diagnostic groups and with a continuous self-report measure of bipolarity using the Hypomania Checklist (HCL-33).

**Results:**

Whilst a trend towards a reduction in the total daily salivary cortisol output—area under the curve with respect to the ground (AUCg)—was detected in depressive disorders across diagnosis, the self-administrated bipolarity index suggested that an increase in bipolarity symptoms predicted lower cortisol levels using AUCg. Chronic cortisol measurement did not discriminate unipolar from bipolar depression.

**Conclusion:**

Results suggested that whilst a low total daily salivary cortisol output (AUCg) might be associated with depressive symptoms, a self-reported measure of bipolarity predicts lower daily cortisol output.

## Introduction

Early detection of bipolar depression can be clinically challenging as major depression is the commonest first clinical presentation for both unipolar and bipolar disorders. Especially in the case of bipolar type II disorder, symptoms can be difficult to detect based on their relatively shorter duration and propensity to lesser intensity than bipolar type I. Furthermore, collateral information can be difficult to obtain in many clinical settings in case of sub-threshold symptoms (Angst 2006).

The crude application of diagnostic criteria based on the unipolar vs. bipolar dichotomy can frequently result in underreporting of bipolar disorder, potentially worsening clinical outcome as the treatment for the two conditions differs (Angst et al. 2011). Although it is clearly important to achieve correct syndromic differentiation, not least to optimise treatment and clinical outcome (Sharma et al. 2005), the absence of salient clinical features to guide differentiation of unipolar from bipolar depression is largely part of the current diagnostic limitations (Benazzi et al. 2002). A relatively new concept is to consider bipolarity as a spectrum on a continuum which allows sub-threshold symptoms to be considered as clinically relevant (Akiskal and Pinto 1999). Method of assessment is also important. For example, Jabben et al. (2011) found that among their large depressive sample, only 5.5% of them were bipolar patients when diagnosed through clinical assessment, but when self-reported (hypo)manic symptoms before the depressive episode were also considered, the bipolar group accounted for 10.5% of the depressive sample.

Another aid to help to differentiate affective disorders is the possibility to utilise putative biological markers to increase the specificity of illness detection. A large amount of evidence suggests that differences in cortisol levels might help correctly identify depressive syndromes (Rybakowski and Twardowska 1999). Hypercortisolemia is most commonly associated with unipolar major depression (Pariante and Lightman 2008), often in the context of severe presentations (Maes et al. 1994). Some evidence also points towards relative peripheral hypocortisolism in bipolar depression (Maripuu et al. 2014) in the presence of treatment resistance (Markopoulou 2013). However, although hypothalamic pituitary adrenal axis abnormalities in affective disorders are widely reported (Daban et al. 2005), the direction of the abnormalities in unipolar vs. bipolar major depression is not clearly defined. Jabben and colleagues recently demonstrated that bipolar spectrum patients had a higher diurnal cortisol slope in saliva measures compared to patients with unipolar major depressive disorder (Jabben et al. 2011). Nonetheless, other reports have shown no alterations in cortisol levels in this condition (Strickland and Percival. 2002; Ciufolini et al. 2014). Furthermore, cortisol levels have not differed when large meta-analyses have studied these alterations in unipolar and bipolar depression (Belvederi Murri et al. 2014, 2016).

The heterogeneity of major depressive disorders is a likely contributor to the lack of homogeneity in the findings. One other possible explanation for the inconsistencies in the literature is the wide variance of the methods used to assess cortisol concentrations, some providing acute measures, others chronic.

A study combining acute and chronic measures by using a viable validated approach to test cortisol concentrations in a relatively homogeneous sample of unmedicated unipolar and bipolar major depression might prove helpful to disentangle this uncertainty. Hair can be used to reliably sample chronic cortisol concentrations in humans (Russell et al. 2012), whereas the measurement of cortisol in saliva samples is a well-established approach to evaluate acute concentrations and variations in levels. Using such an approach previously, we found, for instance, that atypical depression may be better described by alterations in cortisol rhythm, rather than total concentration, when short and long-term cortisol levels were concurrently collected in 1 day (Herane-Vives 2018). This suggestion arose from our finding that patients with atypical depression had normal hair cortisol concentrations (HCC) but reduced daily output measured using salivary cortisol. Some authors have also found that atypical depression is the most frequent form of depressive episode in bipolar disorder patients (Benazzi 2001). By contrast, the large percentage of our depressive sample that did not exhibit any acute or chronic cortisol level alterations was mainly characterised by non-atypical cross-sectional and unipolar longitudinal features.

To date, few case–control studies that studied hair cortisol concentration in affective disorders (Dettenborn et al. 2012; Wei et al. 2015; Dowlati et al. 2010; Hinkelmann et al. 2013; Herane Vives et al. 2015). Among those that have specifically studied HCC in bipolar patients, Aas et al. (2019) found that patients in a current mood episode had higher HCC levels compared to euthymic patients, but HCC was not significantly different when euthymic and non-euthymic bipolar patients were compared to controls. HCC was also similar for schizophrenia and bipolar disorder patients. Conversely, Streit et al. (2016) found that HCC was higher in bipolar disorder compared to schizophrenia. Staufenbiel and Koenders (2014) and Manenschijn et al. (2012) both found that HCC was not associated with bipolar disorder. Only Streit et al. (2016) and Coello et al. (2019) found that HCC was higher in bipolar patients compared with healthy individuals. However, when hair cortisol data have meta-analytically been integrated, results have not shown the presence of an association between HCC alterations and mood disorders (Stalder et al. 2017). To date, no affective disorder research study has used a combination of hair and saliva measures, and compared unipolar vs. bipolar major depression in medication-free individuals.

In this study, we measured acute and chronic cortisol concentrations in a sample of medication free unipolar and bipolar depressed individuals vs. healthy controls. Another point of interest is to use a continuous measure of self-reported bipolarity, as a bipolarity index, to better represent subthreshold bipolarity symptoms in unipolar and bipolar depression and to reflect variations in cortisol concentrations irrespective of clinical diagnosis.

Although prior data as summarised above were inconsistent, we tested the hypothesis that whereas unipolar depression would be characterised by hypercortisolemia, hypocortisolemia would be associated with bipolar depression. We also explored whether a continuous index of bipolarity would correlate more closely with cortisol alterations than a categorical approach, such that the higher the bipolarity index the lower the measured cortisol concentration. Finally, we were interested in whether there were differences when acute and chronic cortisol levels were assessed in the same patients, similar to our previous findings in patients with atypical depression.

## Methods

### Participants

Participants were recruited in London, UK (77) and Santiago, Chile (34) from public advertisements (Wise et al. 2016) and from local psychological therapy and psychiatric services (Table 1). All participants were screened with the Mini International Neuropsychiatric Interview (MINI, Sheehan et al. 1998) and psychometric scales validated in both Spanish and English languages.

**Table 1:** Clinical and demographic characteristics

	Major depression		Healthy controls	p values
	Unipolar	Bipolar		
Controls, *n*				
Total			40	NA
UK			32	
Chile			8	
Unipolar subtype, *n*				
Total	59			NA
UK	37			
Chile	22			
Bipolar subtype, I/II, *n*				
Total		6/6	NA	NA
UK		5/3		
Chile		1/3		
Atypical/non-atypical depression, *n*	24/35	3/9	NA	0.24^λ^
Mean age [years] (SD)	34 (10.5)	31.2 (10.6)	33.2 (8.9)	0.66
M/F	17/42	6/6	11/29	0.3
Episodes; mean (SD)	2.3 (4.0)	10.2 (18.7)	0 (0)	< 0.01
Admissions; mean (SD)	0.1 (0.4)	0.6 (0.9)	0 (0)	< 0.01
Mean duration of illness [weeks], (SD)	91.6 (132.9)	76.6 (151.1)	0 (0)	< 0.01
HAMD-17; mean (SD)	18.2 (4.6)	13.0 (6.8)	0.3 (0.9)	< 0.01
QIDS; mean (SD)	18.2 (4.2)	15.4 (6.7)	0.5 (1.4)	< 0.01
YMRS; mean (SD)	1.1 (1.4)	1.4 (2.7)	0.1 (0.3)	< 0.01

*NA* not applicable, *HAMD−17* 17-item Hamilton Depression Rating Scales, *QIDS* Quick Inventory of Depressive Symptoms, *YMRS* Young Mania Rating Scale, *SD* standard deviation

^λ^p-value was obtained using Fisher’s exact test

Patients were included if they met axis I DSM-IV criteria for unipolar major depression (59) or bipolar (12) disorder. A severity criterion on the 17-item Hamilton Depression rating scale (HAMD-17, Hamilton 1960) was not imposed for two reasons; first, as the HAMD-17 emphasises more typical symptoms of depression over atypical symptoms (e.g. it does not score hypersomnia or increased appetite but does score insomnia and reduced appetite), using a severity inclusion would have risked biasing recruitment because atypical MDE patients would have needed to be more unwell to meet the same HAMD-17 score; and second, so as to reflect the full range of depressive severity seen in out-patients with a MDE. We did, however, require patients to have a score ≥ 11 to ensure they had clinically significant ongoing depressive symptoms. Patients also needed to be medication free for ≥ 2 weeks (≥ 4 weeks for fluoxetine) and not receiving any psychological intervention at the time of the assessment. Depressive symptoms ratings were evaluated on an independent set of patients and showed high inter-rater-reliability among assessors (intraclass correlation coefficient 0.96, p < 0.01). Self-reported symptoms of depression were assessed using the Quick Inventory of Depressive Symptoms (QIDS, Rush et al. 2003). The longitudinal categorical diagnoses of unipolar major depression or bipolar depression were made using the MINI, and a current diagnosis of mania/hypomania was also excluded using the MINI. The Young Mania Rating scale (YMRS, Young et al. 1978) was also used to explore a wider range of mood elation symptoms, and only those with scores of < 10 were included to avoid including those with a mixed affective state. Historical self-reported hypomanic symptoms were assessed using the 33-item hypomania checklist (HCL-33) (Feng et al. 2016). Atypical depression was defined according to its DSM-5 specifier.

Healthy controls had no current or past psychiatric diagnoses, nor did their first-degree relatives (40). All participants required a minimum of 3 cm hair length for inclusion. Any use of illicit substance in the previous 3 months or any unstable medical condition which could affect data analyses or interpretation were exclusion criteria. Differences in cortisol level covariates for hair and saliva specimens, such as age, gender, menstrual cycle phase, hair washing frequency, different hair treatment, and oral contraceptive use were noted and compared between groups. The relevant local ethics committee approved the research and informed consent was obtained from each participant. All participants received a small compensation for taking part in the research.

### Biological specimens

#### Hair specimens

A trained practitioner collected hair samples of suitable participants. The presence and frequency of any biological confounders and procedures potentially affecting hair cortisol levels were measured, including cosmetic treatments (dyeing, bleaching, permanent straightening or waving), hair washing frequency and oral contraceptive use (Stalder et al. 2017a). Collection procedure and analyses for each participant were standardised according to a strict protocol to produce approximately 3 months of hair growth equivalent to 3-month retrospective assessment of endogenous cortisol production. Cortisol levels were determined using a commercially available competitive ELISA (Salimetrics LLC, USA) and the results expressed in picograms of cortisol per milligram of hair (pg/mg). All hair samples were analysed at Salimetrics Laboratory, Cambridge, UK (http://www.salimetrics.com) (Albermann and Musshoff 2012) (see Additional files 1, 2 for procedural details).

### Saliva specimens

Saliva samples collection was taken at the time of the baseline assessment on a weekday Tuesday to Friday following hair sampling. In a typical test day subjects were asked to provide six saliva samples using plain salivettes (Sarstedt, Leicester, UK) as per Roberts’ protocol (Roberts et al. 2004) and instructions were given in writing at the time of the assessment. Saliva samples were taken (1) immediately after awakening, (2) 30 min after awakening, (3) 60 min after awakening, (4) at noon, (5) at 4 p.m., and (6) at 8 p.m. They were also instructed to avoid extremes in the time of collections (before 6 a.m and after 10 p.m.). Analyses of saliva cortisol concentrations were carried out in the Bethem Royal Hospital, London UK. The area under the curve with respect to the ground (AUCg) was used for calculating the daily cortisol output using the six samples. Two measures of cortisol reactivity in saliva were analysed in this study including the cortisol awakening response (CAR) and the delta of cortisol (DELTA). The CAR was calculated as the area under the curve with respect to increase (AUCi) using the first three-morning saliva samples collected over a 1-h period and the DELTA was defined as the difference between cortisol measured at waking time and the sample taken at 30-min. All cortisol measures were calculated in nanomoles per litre (nmol/l) (see Additional files 1, 2 for procedural details).

### Statistical analyses

Demographics, clinical features and questionnaire measurements were compared with ANOVA or t-test for continuous variables and Chi square or Fisher’s exact test for categorical variables. Differences in cortisol levels in unipolar and bipolar depression and healthy controls were tested with analysis of variance (ANOVA) corrected with Bonferroni post-hoc tests for multiple comparisons. Analysis of covariance (ANCOVA) was used to control for any biological confounder potentially affecting cortisol level measurements among the unipolar and bipolar groups. Correlation analysis was used to control for any biological confounder potentially affecting cortisol levels measurements among the whole group of depressed patients. A regression model was used to evaluate the relationship between the bipolarity index, calculated as the total HCL-33 score, and measures of cortisol concentrations in the whole sample.

## Results

### Subjects

Fifty-nine patients with unipolar major depression (UD) and 12 participants with bipolar depression (BD) (6 type I and 6 type II) were matched with a group of 40 healthy individuals (Table 1). There were no significant differences across groups in terms of confounding biological variables aside from hair washing occurring more frequently in bipolar subjects, and higher use of contraceptives in depressed subjects (Table 2).

**Table 2:** Distribution of biological variables in the samples

	Major depression		Healthy controls	p-value
	Unipolar depression	Bipolar depression		
Mean BMI (SD)	25.9 (4.5)	25.9 (7.2)	24.3 (3.6)	0.22
Mean waist circumference in cm (SD)	87.1 (12.9)	87.2 (17.3)	82.1 (10.7)	0.15
Follicular phase, *n* (%)	12 (20.3)	1 (8.3)	9 (22.5)	0.44
Medication,* n* (%)	22 (37.3)	3 (25)	13 (32.5)	0.70
Contraceptives, *n* (%)	3 (5.1)	2 (16.6)	10 (25)	0.04
Medical comorbidities, *n* (%)	12 (20.3)	2 (16.7)	2 (5.0)	0.07
Mean hair washes/week (SD)	3.9 (1.7)	5.7 (2.1)	4.5 (1.7)	0.02
Cosmetic treatments^a^, *n* (%)	33 (55.9)	9 (75)	27 (67.5)	0.47

*BMI* body mass index, *NA* not applicable, *SD* standard deviation

^a^Dyeing, bleaching, permanent straightening or waving

### Hair cortisol concentrations

Analyses of hair cortisol concentration in the combined group of major depression (N = 71) (8.3, SD 4.6 pg/mg) vs. healthy individuals (8.3, SD 3.9 pg/mg) did not show significant group differences (p = 0.96). Similarly, hair cortisol concentration in each comparison of UD group (7.9, SD 4.3 pg/mg) vs. BD (10.2, SD 5.3 pg/mg) vs. HC (8.3, SD 3.9 pg/mg) did not show significant results (F (2,108) = 1.32, p:0.27). Analysis of covariance controlling for frequency of washing hair and contraceptive use remained non-significant (F (4,71) = 0.42, p = 0.79).

### Saliva cortisol concentrations

**Fig. 1:**
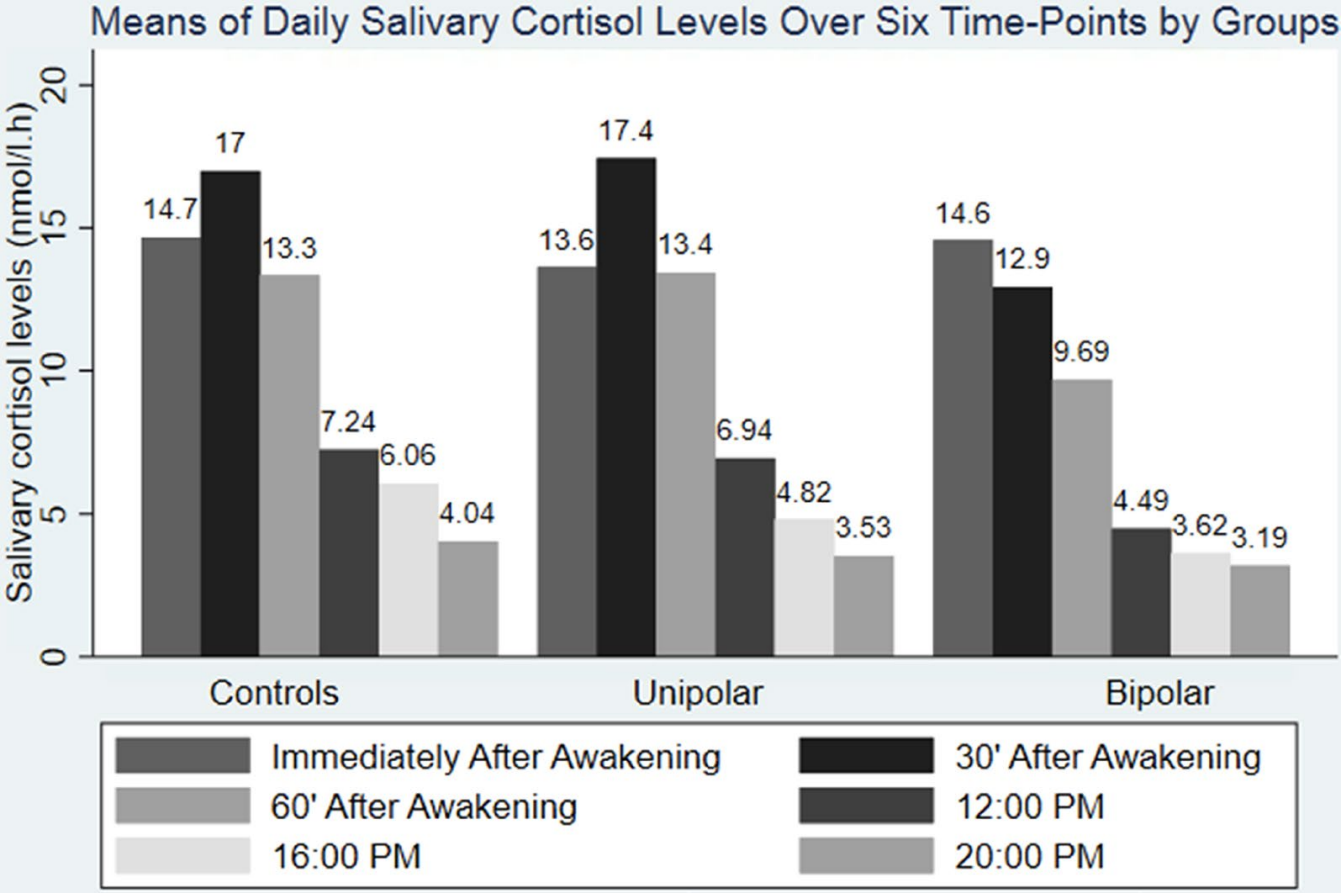
Daily salivary cortisol levels over 6 time-points by groups

There was a trend towards significant reduction in the total daily salivary cortisol output (AUCg) in the combined group of depressed individuals (105.8, SD 34.8 nmol/l h) vs. the healthy controls (120.8, SD 38.9 nmol/l h), p = 0.06 although neither the UD (108.3, SD 34.8 nmol/l h) nor the BD group (87.8; SD 32.0 nmol/l h) significantly differed from the healthy individuals (all p > 0.05). Correlation analysis between the use of contraceptives and AUCg or HCC did not show a significant association (r = 0.11; p = 0.35 and r = 0.02; p = 0.83, respectively). Neither CAR nor DELTA of cortisol showed a significant difference in any of the comparisons (all p > 0.05) (See Fig. 1 for daily salivary cortisol levels over 6 time-points by groups).

### Relationship between saliva, hair cortisol concentrations and self-measure of bipolarity in the whole sample

**Fig. 2:**
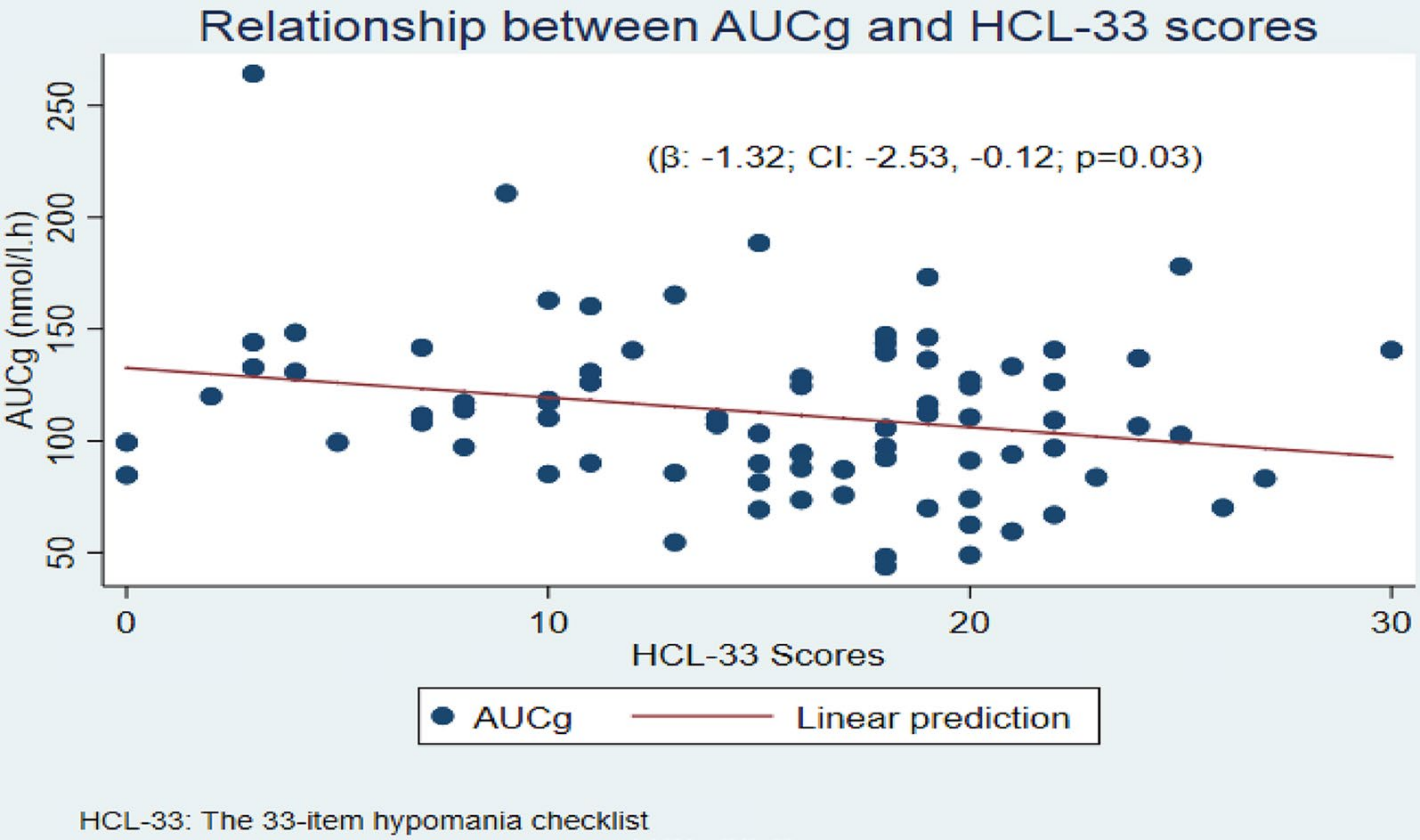
Relationship between AUCg and HCL-33 scores

A linear regression carried out to investigate the relationship between cortisol and the bipolarity index as a continuum in the whole sample indicated a significant negative correlation between AUCg and HCL-33 score (β − 1.32; CI − 2.53, − 0.12; p = 0.03). On average AUCg decreased 1.32 nmol/l h for every point increase in the HCL-33 scale (Fig. 2). Regression analyses between HCL-33 scores and CAR (β − 0.05, p = 0.58, CI − 0.25, − 0.14) or DELTA of cortisol (β 0.01, p = 0.73,CI − 0.09, − 0.13) were not significant (Fig. 2). No significant relationship was found with hair cortisol concentrations (β 0.02; CI − 0.09, 0.15; p = 0.64).

## Discussion

In this work, we set out to measure acute and chronic cortisol concentrations in major unipolar and bipolar depression. We also indexed bipolarity in the whole sample, measured using a self-rated scale, to test whether this approach might increase the power to identify predictable changes in cortisol concentrations irrespective of clinical diagnosis. Whereas acute and chronic cortisol concentrations did not significantly differ across clinically defined groups, a linear pattern of cortisol variation emerged so that a higher self-reported bipolarity index was associated with lower AUCg.

We did not find hypercortisolaemia in unipolar major depression as we postulated either using hair or saliva measures. This is different from studies by Dettenborn et al. (2012) and Wei et al. (2015) that showed higher cortisol concentration in this group of patients compared to healthy controls. In the studies by Dettenborn et al. (2012a, b) patients were medicated while Wei et al. (2015) studied unipolar unmedicated patients who were experiencing their first depressive episode. Their findings suggest that that hypercortisolaemia is a frequent finding in more severe patients and suggest that hypothalamic pituitary adrenal axis responses might be increased at first presentation. The patients included in the present study were not purely first presenters which in combination with other factors (e.g. different subtypes of depression such as atypical and non-atypical) add additional heterogeneity and might explain the finding of an absence of hypercortisolaemia.

When major depression was considered irrespective of clinical diagnosis, hair cortisol concentrations did not differ from healthy controls. There was however a trend towards lowered daily cortisol output in this group. Although this was not a significant finding (confirmed after controlling for biological confounders), the direction is different than the hypercortisolaemia suggested in other work (Pariante and Lightman 2008). The present study measured cortisol output over a period of 12 h. Taking into account the results of our hair cortisol measurements which covered 3 months, it is possible that the presence of large spikes of hypercortisolaemia on some days but not others create an average chronic normocortisolaemia as reflected in hair. The contrast between acute and chronic measurements might, therefore, suggest an intermittently hyperactive pattern of cortisol secretion in major depression or perhaps a compensatory increase in cortisol levels during the night, which would be undetected when measuring day time cortisol output, as suggested in some studies (Krieger et al. 1971; Hellman et al. 1970). Other salivary measurements, such as those related to cortisol reactivity e.g. CAR and DELTA of cortisol, were not discriminative in this study.

One of the reasons for hypothesising there might be hypocortisolaemia in bipolar depression was the anticipated frequency of atypical depression. Previously, Benazzi (2001) showed that atypical depression was the most frequent type of depressive episode related to bipolar disorder. However, atypical depressive episodes were not the most frequent cross-sectional depressive subtype in our bipolar sample (33%). Furthermore, only half of our sample were diagnosed with Bipolar II disorder (see Table 1), which according to (Benazzi 2001) is the subtype most frequently associated with atypical episodes. Whilst bipolar subtypes may have different chronic cortisol secretion profiles, our sample of bipolar patients was too small to allow meaningful comparison of cortisol concentrations between Bipolar I and Bipolar II.

Overall, our results are then more closely related to Staufenbiel and Koenders (2014) and Manenschijn et al. (2012) who both found normocortisolaemia in hair in a similar studies in samples of bipolar subjects.

However, conversely to results from Girshkin et al. (2014) our linear regression model indicated that a short term measure of daily salivary cortisol output, the AUCg, was the only saliva measure that acted as an explanatory variable when HCL-33 scores were used to define bipolarity dimensionally in the whole sample. The model showed that increases in the HCL-33 scores were significantly associated with a decrease in cortisol levels in the AUCg. This finding suggests a significant inverse relationship between average AUCg and HCL-33 score.

One of the main limitations of this study is that, although the majority of the work to date supports the use of hair to measure cortisol levels (Kalra et al. 2007; Van Uum et al. 2008; D’Anna-Hernandez et al. 2011; van Holland et al. 2012; Grass et al. 2015), there is still some uncertainty on the validity of this method (e.g. Sharpley 2012). In addition, while hair growth differences have been found in white, black and Asian ethnicities (Loussouarn et al. 2005), there is no report to date supporting or discounting differences between English and Chilean people. There is also uncertainty concerning cosmetic treatments, which are known to affect measurement taken from the outside of the hair (Sauvé et al. 2007; Manenschijn et al. 2011) although we measured cortisol from inside of the shaft (see Additional files 1, 2). We also assumed the irrelevance of other factors such as body sweat, shown not to affect cortisol measurements (Grass et al. 2015), and washout effects known to be relevant with hair lengths above 4 cm (Dettenborn et al. 2010). Another limitation is that a new protocol regarding CAR assessment has recently been published. It recommends routinely assessing CAR on two consecutive days, rather than on 1 week day (Stalder et al. 2016), which was not the case in the current study. Finally, we cannot generalize our result to the large percentage of the population that has less than 3 cm of hair length.

In conclusion, we did not find statistically significant differences in patterns of secretion of cortisol measured by using saliva and hair samples between unipolar and bipolar depression. However, self-reported bipolarity measures may be more dimensionally linked to short-term cortisol output variations.

## Supplementary information

**Additional file 1.** Hair collection procedures.

**Additional file 2.** Saliva specimen collection procedures.

## Data Availability

The datasets generated and/or analysed during the current study are not publicly available due ongoing research projects but are available from the corresponding author on reasonable request. AHV had AJC had full access to all of the data in the study and take responsibility for the integrity of the data and the accuracy of the data analysis.
