# Supplementary material for: Cortisol levels in unmedicated patients with unipolar and bipolar major depression using hair and saliva specimens
Source: Int J Bipolar Disord. 2020 Mar 5;8:15. doi: 10.1186/s40345-020-0180-x (PMC7056775; doi:10.1186/s40345-020-0180-x)
Supplement: Supplementary file 1 — Additional file 1. Hair collection procedures. [file 40345_2020_180_MOESM1_ESM.docx]

**Additional File 1: Hair collection procedures**

**A) Collection**

Hair samples were taken from the vertex at the back of the head and were cut with sterilised scissors as close to the scalp as possible. For this study, four locks of hair were required from different places from the vertex posterior, each to be the approximate thickness of a rubber band of 1 centimetre. At the laboratory, 3 cm of hair measured from the end to the scalp surface were cut from each lock, representing approximately 3 months of hair growth equivalent to 3-month retrospective assessment of cortisol production. The total weight of the four 3 cm segments from each lock is approximately equivalent to 60-80 mg of hair. Once collected, hair samples were stored at room temperature in the dark in a sealed container.

**B) Analysis**

Prior to analysis, the hair samples were washed in 1 ml of isopropanol to remove external contaminants, the isopropanol was removed from the vial and the hair allowed to dry in a clean air environment for 48 hours. Once fully dry five ceramic balls were added to each tube and the hair samples ground to a powder using am Fast Prep-24 (MP Biomedicals, LLC). To extract cortisol, 1.75 ml of methanol was added to each sample and the samples incubated for 20 hours whilst rotating the samples constantly.

The hair, methanol and ceramic balls were decanted into a polypropylene tube (Sarstedt AG & Co, Germany) that separated the ceramic balls from the rest of the mixture. The tube was centrifuged at 3000 RCF to separate the ground hair and methanol and 1.25ml of the clear methanol supernatant was decanted into a 2ml polypropylene cryovial. The methanol was then removed using a vacuum centrifuge (Scan Speed 40, Labgene) and the tubes frozen at -80°C until required for the cortisol ELISA. Cortisol levels were determined using a commercially available competitive ELISA (Salimetrics LLC, USA). Samples were thawed and reconstituted with 0.125ml of Salimetrics cortisol assay diluent and the samples were then assayed in accordance with the manufacturer’s protocol. The results were expressed as picograms of cortisol per milligram of hair. All hair samples were analysed at Salimetrics Laboratory, Cambridge, UK (www.salimetrics.com).
