# Supplementary material for: Cortisol levels in unmedicated patients with unipolar and bipolar major depression using hair and saliva specimens
Source: Int J Bipolar Disord. 2020 Mar 5;8:15. doi: 10.1186/s40345-020-0180-x (PMC7056775; doi:10.1186/s40345-020-0180-x)
Supplement: Supplementary file 2 — Additional file 2. Saliva specimen collection procedures. [file 40345_2020_180_MOESM2_ESM.docx]

**Additional File 2: Saliva specimen collection procedures**

**A) Collection**

Subjects were instructed not to smoke, brush their teeth, or have anything to eat or drink for at least an hour before the collection of the samples. Samples were collected (1) immediately after awakening, (2) 30 minutes after awakening, (3) 60 minutes after awakening, (4) at noon, (5) at 4 pm, and (6) at 8 pm. Participants were instructed to avoid collections before 6 am and after 10 pm to minimise confounders. All participants filled out a questionnaire on socio-demographic details (gender, smoking habits, and health problems). Subjects were also instructed to specify whether they experienced any stressors and to provide any information which could be of relevance and/or interfere with the study. Moreover, they were asked to note the exact time for each saliva sample in a research log to assess self-reported compliance. Subjects were instructed to storage and delivery the research team.

**B) Analysis**

Analyses of saliva cortisol concentrations were carried out in the Bethem Royal Hospital, London UK. On the arrival to the laboratory, the salivettes were frozen at −20 ° Celsius. After thawing, they were centrifuged at 3500 rev/min for 10 min, which resulted in a clear supernatant of low viscosity. The saliva specimens were then frozen again in microtubes. Saliva cortisol concentrations were subsequently determined using the “Immulite” —DPC’s Immunoassay analyser (www.diagnostics.siemens.com). To plot a calibration graph, set of 22 cortisol standards in saline were used in each assay. Results were highly reproducible with a mean slope of 0.197 and standard error of the mean (SEM) ± 0.004 and the method correlated well with a previously published Time-Resolved fluorescence immunoassay (TR-FIA) (Mondelli et al. 2010). It had analytical sensitivity of 0.2 nmol/l.h and inter/intra assay precision- total imprecision in percentage (% CV) was less than 10% (cortisol concentration range 5 to 25 nmol/l.h)-. All samples from the same subject were analysed in the same run.
